# Transgenic Rat Model of Neurodegeneration Caused by Mutation in the *TDP* Gene

**DOI:** 10.1371/journal.pgen.1000887

**Published:** 2010-03-26

**Authors:** Hongxia Zhou, Cao Huang, Han Chen, Dian Wang, Carlisle P. Landel, Pedro Yuxing Xia, Robert Bowser, Yong-Jian Liu, Xu Gang Xia

**Affiliations:** 1 Department of Pathology, Anatomy, and Cell Biology, Thomas Jefferson University, Philadelphia, Pennsylvania, United States of America; 2 Center for Biotechnology, University of Nebraska–Lincoln, Lincoln, Nebraska, United States of America; 3 Department of Microbiology and Immunology, Thomas Jefferson University, Philadelphia, Pennsylvania, United States of America; 4 Lower Merion High School, Ardmore, Pennsylvania, United States of America; 5 Department of Pathology, Center of ALS Research, School of Medicine, University of Pittsburgh, Pittsburgh, Pennsylvania, United States of America; 6 Department of Neurobiology, School of Medicine, University of Pittsburgh, Pittsburgh, Pennsylvania, United States of America; The Jackson Laboratory, United States of America

## Abstract

TDP-43 proteinopathies have been observed in a wide range of neurodegenerative diseases. Mutations in the gene encoding TDP-43 (i.e., *TDP*) have been identified in amyotrophic lateral sclerosis (ALS) and in frontotemporal lobe degeneration associated with motor neuron disease. To study the consequences of *TDP* mutation in an intact system, we created transgenic rats expressing normal human *TDP* or a mutant form of human *TDP* with a M337V substitution. Overexpression of mutant, but not normal, *TDP* caused widespread neurodegeneration that predominantly affected the motor system. *TDP* mutation reproduced ALS phenotypes in transgenic rats, as seen by progressive degeneration of motor neurons and denervation atrophy of skeletal muscles. This robust rat model also recapitulated features of TDP-43 proteinopathies including the formation of TDP-43 inclusions, cytoplasmic localization of phosphorylated TDP-43, and fragmentation of TDP-43 protein. *TDP* transgenic rats will be useful for deciphering the mechanisms underlying TDP-43–related neurodegenerative diseases.

## Author Summary

Amyotrophic lateral sclerosis, a condition also known as Lou Gehrig's disease, is characterized by progressive degeneration of motor neurons, denervation atrophy of skeletal muscles, and eventual paralysis of affected limbs. The signature pathology of Lou Gehrig's disease is the formation of intracellular inclusions containing phosphorylated TDP-43 protein. Most cases of Lou Gehrig's disease do not have a clear cause, while only about 10% of the cases are caused by mutation of individual genes. Here, we describe a novel rat model that expresses a mutated form of the human gene encoding TDP-43 and manifests the phenotypes and pathological features observed in patients with Lou Gehrig's disease. Laboratory rats are the preferred animals for pharmacological studies. Therefore, this new rat model will be useful not only for mechanistic study of Lou Gehrig's disease, but also for the development of therapies for this devastating disease.

## Introduction

TAR DNA-binding protein (TDP-43) is a highly conserved ribonucleoprotein that is encoded by the *TDP* gene and can bind to RNA, DNA, and proteins [1]-[3]. In mammals, the primary transcript of the *TDP* gene can be alternatively spliced to generate 11 mRNA molecules. The major splice variant is full-length and encodes TDP-43 [4]. While the functions of this complex molecule remain largely unknown, ubiquitinated and phosphorylated TDP-43 accumulates in the nucleus and cytoplasm of affected cells in sporadic amyotrophic lateral sclerosis (ALS) and frontotemporal lobe degeneration (FTLD) [5],[6]. TDP-43 resides predominately in the nucleus and its translocation to the cytoplasm appears to be an early event in the pathological process underlying sporadic ALS [7]. At the end-stages of sporadic ALS and FTLD, C-terminal fragments of TDP-43 are remarkably increased in the brain [5],[6], but the full-length protein remains the major species in spinal cord [8], suggesting that regional differences exist in the metabolism and pathological mechanisms of TDP-43. Although TDP-43 proteinopathies have been identified in a wide range of neurodegenerative diseases including sporadic ALS, FTLD, Alzheimer's disease, and dementia with Lewy bodies [5]–[9], TDP-43 inclusions have not been detected in familial ALS caused by mutation of the *SOD1* and *FUS* genes [10]–[13]. These findings imply that TDP-43 proteinopathy is common to neurodegenerative diseases and that divergent pathological processes may underlie sporadic and familial cases of ALS.

Mutations in the *TDP* gene segregate with ALS and FTLD associated with motor neuron disease (FTLD-MND) in geographically unrelated families [14]–[18], suggesting that *TDP* mutation is pathogenic in a subset of neurodegenerative diseases. Transient expression of the mutant, but not the normal, human *TDP* gene leads to apoptotic death of spinal motor neurons in chicken embryos [15]. In *Drosophila melanogaster*, depletion of the *TDP* homolog results in deficient locomotor activity and defects at neuromuscular junctions (NMJs) [19]. Suppression of *TDP* gene expression induces cell death in cultured neuroblastoma cells [20]. Previous studies indicate that mutation of the *TDP* gene is neurotoxic and that normal TDP-43 is important to cellular function; however, how mutations in the *TDP* gene cause neurodegeneration remains unknown.

To study the consequences of *TDP* mutation in an intact system, we expressed a mutant form of the human *TDP* gene in rats, which were chosen over mice because they are the preferred animals for pharmacological studies. Overexpression of a mutant, but not the normal, human *TDP* gene caused widespread neurodegeneration, which predominantly affected the motor system. Transgenic rats that constitutively or conditionally expressed a mutant form of human *TDP* with a valine-to-methionine substitution at position 337 (M337V) developed similar phenotypes at early ages, the phenotypes that were characterized by motor neuron degeneration accompanied by astrocyte and microglial activation in the spinal cord.

## Results

### Constitutive expression of a mutant, but not the normal, human *TDP* gene causes early death in transgenic founder rats

**Figure 1:**
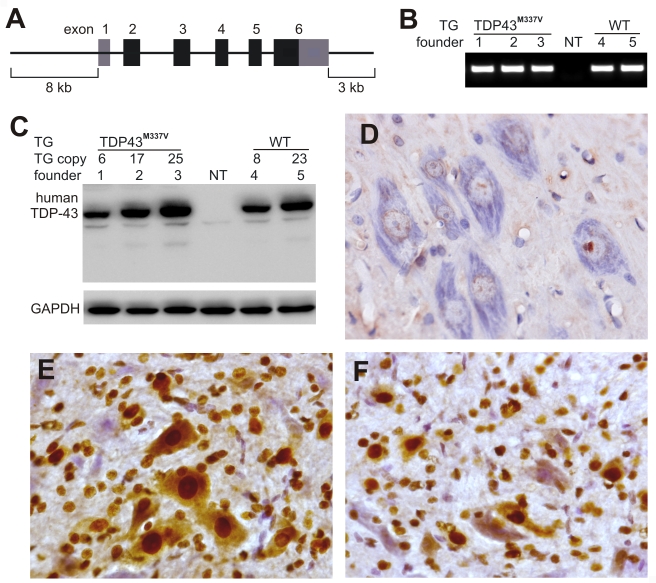
Postnatal death in transgenic founder rats constitutively expressing a mutant human *TDP* transgene. (A) Schematic diagram shows the structure of the human *TDP* transgene extracted from a BAC clone. The M337V mutation was introduced into the *TDP* transgene construct using a recombineering technique. (B) PCR identified transgenic founders carrying the normal (WT) or mutant (TDP43^M337V^) human *TDP* gene. NT: nontransgenic littermate. (C) Immunoblotting detected a robust expression of the *TDP* transgene in the forebrain of TDP43^M337V^ transgenic founders and TDP43^WT^ transgenic offspring (first generation). Membranes were probed with an antibody against human TDP-43 (generated in-house) and then with an antibody against GAPDH. NT: a nontransgenic littermate. (D–F) Immunohistochemistry revealed that human TDP-43 was expressed in the normal (E) and mutant (F) human *TDP* transgenic rats, but not in nontransgenic littermates (D) at the age of 29 days. Transverse sections through the L4 spinal cord were stained for human TDP-43 immunoreactivity and then counterstained with haematoxylin. Micrographs show the ventral horn of the lumbar spinal cord.

TDP-43 is widely expressed in mammalian tissues [21]. To mimic the expression profile of the endogenous *TDP* gene, we extracted the minimal human *TDP* gene (mini *TDP* gene) from a BAC clone and discarded the excess flanking sequences. The mini human *TDP* transgene contains essential elements for regulating transgene expression but does not carry unwanted genes into transgenic rats (Figure 1A). Among all known mutations in the *TDP* gene, the M337V substitution is found in geographically unrelated families and thus is an excellent representative of *TDP* gene mutations [15],[17]. We introduced the M337V mutation into the mini TDP transgene using a recombineering technique [22]. Using pronuclear injection, we generated three transgenic founders (two males: founders 1 and 2; one female: founder 3) that robustly expressed the miniTDP43^M337V^ transgene (Figure 1A–1C and 1F). The mutant *TDP* transgenic founders were indistinguishable from their nontransgenic littermates at birth; however, they soon lost mobility and died at postnatal ages. Founder 3 died at the age of 10 days. Founder 2 showed weakness in the limbs at the age of 13 days and became paralyzed by the age of 18 days. Founder 1 showed weakness in a forelimb at the age of 21 days and became paralyzed by the age of 29 days. We examined founder 1 using immunohistochemistry and observed a reduction in motor neurons in the ventral horn of the lumbar spinal cord (Figure 1F). Since none of the mutant *TDP* (miniTDP43^M337V^) transgenic rats survived to sexual maturity, mutant *TDP* transgenic lines could not be established. In parallel, we generated two transgenic founder rats that carried the normal human *TDP* transgene (miniTDP43^wt^), which had an identical DNA composition as miniTDP43^M337V^ except that it lacked the M337V mutation (Figure 1A–1E). The miniTDP43^wt^ transgenic rats expressed human TDP-43 protein at levels comparable to those detected in the miniTDP43^M337V^ transgenic founder rats but did not develop paralysis by the age of 200 days. These findings suggest that the disease phenotypes observed in the miniTDP43^M337V^ transgenic founder rats result from toxicity of the *TDP* gene mutation.

### Temporal expression of a mutant human *TDP* gene in postnatal rats causes progressive paralysis

**Figure 2:**
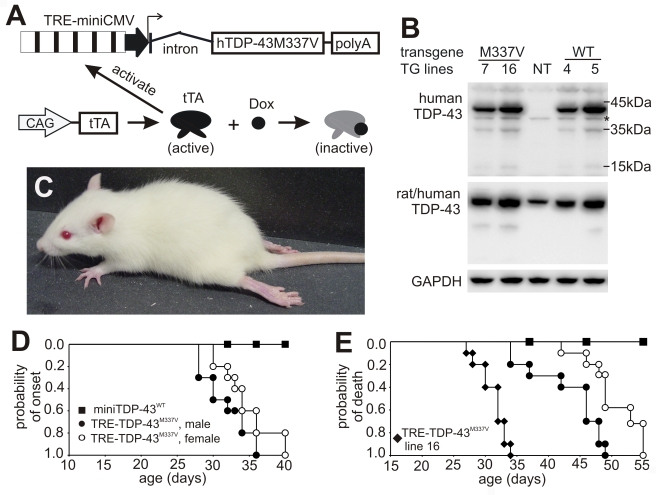
Progressive paralysis in transgenic rats conditionally expressing a mutant human *TDP* gene. (A) Schematic diagram shows the structure of the inducible mutant *TDP* transgene (TRE-TDP-43^M337V^). Expression of the mutant *TDP* transgene depends on tTA activation and can be suppressed by Dox, which binds to tTA and renders it inactive. (B) Immunoblotting detected a robust expression of the transgene in the spinal cord of P20 rats. Membranes were probed sequentially with antibodies against human TDP-43 (generated in-house), human and rat TDP-43 (ProteinTech), and rat GAPDH. M337V: transgenic rats carrying the conditional mutant *TDP* (TRE-TDP-43^M337V^) and CAG-tTA transgenes; WT: transgenic rat carrying the mini normal human *TDP* transgene (miniTDP-43^WT^); NT: nontransgenic littermate of the WT transgenic rat; *, a weak nonspecific band. (C) Photo of a mutant *TDP* transgenic rat (line 7) paralyzed at P40. (D) Graphs show the probability of disease onset, defined as an unrecoverable reduction in running time on a Rotarod. Disease onset for line 16 was not plotted, since these animals experienced early paralysis with rapid progression and accurate definition of disease onset was technically difficult. (E) Survival analysis revealed that lifespan was remarkably reduced in the mutant *TDP* transgenic rats. Rats were euthanized and counted as dead when two or more legs became paralyzed. Male and female rats of line 16 were combined as one group because disease progression between each gender was indistinguishable. Definition of symbols in (D,E): **▪**, normal *TDP* transgenic rats carrying the miniTDP-43^WT^ transgene (line 4; n = 9); • and ○, mutant male (•, n = 10) and female (○, n = 12) transgenic rats carrying the TRE-TDP-43^M337V^ (line 7) and CAGtTA transgenes; **♦**, mutant *TDP* transgenic rats carrying the TRE-TDP-43^M337V^ (line 16) and the CAGtTA transgenes (n = 14). All breeding female rats were given Dox in drinking water (50 µg/ml) until 4 days before delivery.

Since constitutive expression of a mutant human *TDP* gene caused a severe phenotype in transgenic founders, we used a tetracycline (Tet) regulatory system to express the mutant *TDP* transgene in a controlled manner. In this way, we could establish transgenic rat lines expressing the human *TDP* transgene with a pathogenic mutation. The Tet-off system is commonly used in transgenic studies and is comprised of only two elements— a Tet-controlled transactivator (tTA) and a tTA-activated promoter (TRE) [23]. Using pronuclear injection, we established two transgenic lines (line number corresponds to transgene copy) that carry 7 or 16 copies of the TRE-TDP-43^M337V^ transgene under the control of the TRE promoter (Figure 2A). The transcriptional activator, tTA, is inactive in the presence of the Tet derivative, Doxycycline (Dox), allowing for inactivation of a TRE promoter-controlled gene through Dox administration in the bigenic rats that carry the TRE-TDP-43^M337V^ and the tTA transgenes (Figure 2A). In the absence of Dox, tTA constantly activates the TRE-TDP-43^M337V^ transgene, producing an expression pattern that is indistinguishable from constitutive transgene expression [24].

Constitutive expression of the miniTDP-43^M337V^ transgene caused postnatal death in the transgenic founder rats (Figure 1), suggesting that the mutant *TDP* gene is highly toxic. To test whether the severe phenotype observed in the constitutive transgenic rats could be reproduced in conditional transgenic rats, we produced the TRE-TDP-43^M337V^ and tTA double transgenic rats by crossing the TRE-TDP-43^M337V^ transgenic lines with a tTA transgenic line that expresses the tTA transgene at levels sufficient to vigorously activate tTA reporter genes [24]. To obtain a constitutive pattern of transgene expression, we allowed the TRE-TDP-43^M337V^ transgene to be expressed from early embryogenesis by withholding Dox treatment. Consistent with findings in constitutive transgenic rats (Figure 1), expression of the TRE-TDP-43^M337V^ transgene from early embryonic stages caused severe phenotypes in the conditional transgenic rats of line 16 (Figure 2B). Transgenic rats of line 16 became paralyzed and died by postnatal day 20 (P20). The similarity in phenotypes between the constitutive and conditional transgenic rats indicates that the observed defects did not result from an insertional mutation.

Expression of the TDP-43^M337V^ transgene from early embryogenesis caused early death in transgenic rats, making functional analysis of this model a challenge. To facilitate analysis of motor function, we added Dox to the drinking water (50 µg/ml) of breeding rats to suppress transgene expression during embryonic development. We then withdrew Dox at 4 days before delivery to allow for recovery of transgene expression in postnatal rats. As a result, the transgene was not expressed in newborn pups but was fully expressed in postnatal rats by P10 (Figure S1). The TRE-TDP-43^M337V^ transgenic rats of line 16 showed a rapid progression of disease phenotypes, exhibiting limb weakness by P20 and paralysis before P35 (Figure 2E). In contrast, the TRE-TDP-43^M337V^ transgenic rats of line 7 showed a later onset and a slower progression of similar phenotypes (Figure 2C–2E). Disease progression in line 7 could be divided into four distinct stages [25]: the nonsymptomatic stage, disease onset, the paralysis stage, and the disease end stage. Disease onset was defined as an unrecoverable reduction in running time on a rotating Rotarod. The paralysis stage was defined as visible dragging of a limb. The disease end stage was defined as paralysis in two or more limbs. Postnatal rats aged 21 days were subjected to a Rotarod test to determine disease onset (Figure 2D). Since transgenic rats of line 16 developed early paralysis and had a rapid disease progression, determining the time of disease onset for this high-copy line was technically difficult. Transgenic rats of line 16 showed limb weakness by an age of 20 days and became paralyzed in the legs by an age of 35 days, with no sexual dimorphism existing in the rate of disease progression (Figure 2E). In contrast, transgenic rats of line 7 displayed sexual dimorphism in the time of disease onset and in the rate of disease progression (Figure 2D and 2E). Sexual dimorphism in phenotypic onset has also been observed in an ALS animal model expressing mutant human *SOD1* genes [26]–[29]. The disease phenotypes observed in the mutant *TDP* (TRE-TDP-43^M337V^) transgenic rats were not observed in normal *TDP* transgenic rats (miniTDP-43^WT^) by an age of 200 days, though these rats expressed the human *TDP* transgene at comparable levels as TRE-TDP-43^M337V^ rats (Figure 2B–2E). An examination of TRE-TDP-43^M337V^ transgenic offspring revealed that, consistent with findings in miniTDP-43^M337V^ transgenic founders (Figure 1), the disease phenotypes in these animals were related to mutation of the *TDP* gene.

### Axon terminals are the primary targets of degeneration caused by mutation of the *TDP* gene

**Figure 3:**
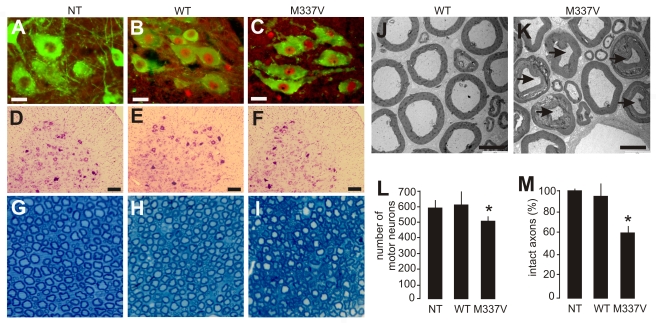
Neuronal death and axonal damage in transgenic rats expressing a mutant human *TDP* gene. (A–C) Transverse sections of lumbar spinal cord were immunostained for human TDP-43 (red) and ChAT (green). Scale bars: 30 µm. (D–F) Cresyl violet staining revealed motor neurons in the ventral horn of L3 spinal segments. Scale bars: 100 µm. (G–I) Toluidine blue staining shows axons of L3 ventral roots. (J,K) Transmission electron microscopy (EM) shows the structure of axons in the L3 ventral roots. Degenerating axons were shrunken and had collapsed myelin (arrows). Scale bars: 5 µm. (L) The number of large neurons (>25 µm in diameter) in the ventral horn of L3 spinal segments was estimated by stereological cell counting. Data are expressed as the mean ± SD (*n* = 9–15). *p<0.05. (M) Axons of L3 ventral roots were visualized by EM, and 60 axons (>4 µm in diameter) of each animal were examined for integrity. Data are expressed as mean ± SD (*n* = 8 or 9). *p<0.01. Tissues were collected from paralyzed transgenic rats of line 7 (age: 45±7 days) conditionally expressing the mutant human *TDP* gene (M337V: C, F, I, K, L, and M), age-matched nontransgenic littermates (NT: A, D, G, L, and M), and age-matched transgenic rats constitutively expressing the normal human mini *TDP* gene (WT: B, E, H, J, L, and M).

**Figure 4:**
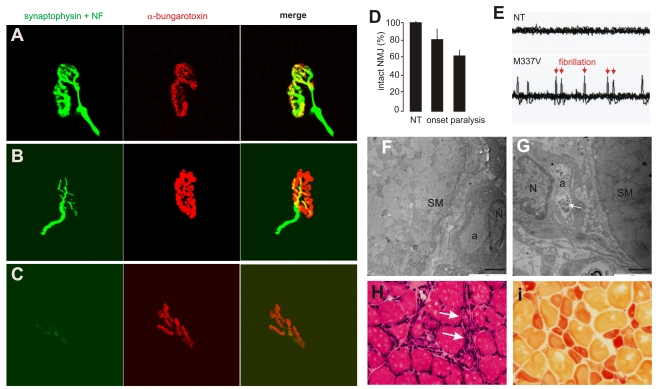
Denervation atrophy of skeletal muscle in transgenic rats expressing a mutant human *TDP* gene. (A–C) The neuromuscular junction (NMJ) in the gastrocnemius muscles was examined by confocal microscopy to reconstruct the focal structure. Compared to the NMJ in a control rat (A), the NMJ in TRE-TDP-43^M337V^ transgenic rats (line 7) was partially denervated at disease onset (B) and severely denervated at disease end stages (C). Axon terminals were visualized by immunostaining for synaptophysin and neurofilament (NF), while postsynaptic nicotinic receptors were visualized with Alexa fluor 555-conjugated α-bungarotoxin. (D) Quantification of NMJ denervation in nontransgenic control rats (NT) and TRE-TDP-43^M337V^ transgenic rats at disease onset or the paralysis stage. Twenty NMJs were examined for each animal (n = 4 or 5). (E) Electromyography of the gastrocnemius muscles revealed that frequent fibrillation potentials (arrows) were present in paralyzed TRE-TDP-43^M337V^ transgenic rats (M337V), but not in a nontransgenic littermate (NT). (F,G) EM revealed that intramuscular axons underwent degeneration in a mutant transgenic rat (line 7, age: 32 days) at disease onset (G), but not in an age-matched nontransgenic littermate (F). The arrow indicates a cluster of aggregates that accumulated in the axon of a mutant rat (G). SM: skeletal muscle; a: axon; N: the nucleus of a Schwann cell. Scale bars: 2 µm. (H,I) Group atrophy of the gastrocnemius muscle in paralyzed TRE-TDP-43^M337V^ transgenic rats was detected by H&E staining (H, arrows) and by histochemistry for nonspecific esterase (I).

Anatomical analysis revealed that motor neurons in the spinal cord robustly expressed the human *TDP* transgene (Figure 3A–3C). The number of spinal motor neurons was significantly reduced in mutant *TDP* transgenic rats but not in normal *TDP* transgenic rats (Figure 3D–3F and 3I), although the mutant and normal *TDP* transgenic rats expressed human TDP-43 at comparable levels (Figure 2B). Large-caliber neurons were preferentially affected in mutant rats at the end stages of disease (Figure 3D–3F and 3L). During the paralysis stage, degenerating axons were clearly visible in the ventral (Figure 3G–3I and 3M) and dorsal roots (Figure S2), with motor axons of the corticospinal track also being affected (Figure S3). Confocal microscopy revealed that denervation of synaptic endplates in skeletal muscle occurred at disease onset (Figure 4B and 4D) and worsened at the end stage of disease (Figure 4C and 4D). Electron microscopy confirmed that, in the mutant transgenic rats, degeneration of motor neuron axons occurred at disease onset (Figure 4F and 4G); however, no loss of motor neurons was detected in the mutant *TDP* transgenic rats at this time. These findings suggest that axon terminals are the primary targets of degeneration associated with pathogenic mutation of *TDP*. In the mutant *TDP* transgenic rats, denervation of skeletal muscle fibers was confirmed by electromyography, which detected frequent fibrillation potentials—a characteristic of muscle denervation and regeneration (Figure 4E). As results of denervation, groups of skeletal muscles were atrophied (Figure 4H and 4I). These pathological changes were correlated with progressive paralysis in the mutant transgenic rats (Figure 2C–2E).

### Neurodegeneration is accompanied by glial reactivity

**Figure 5:**
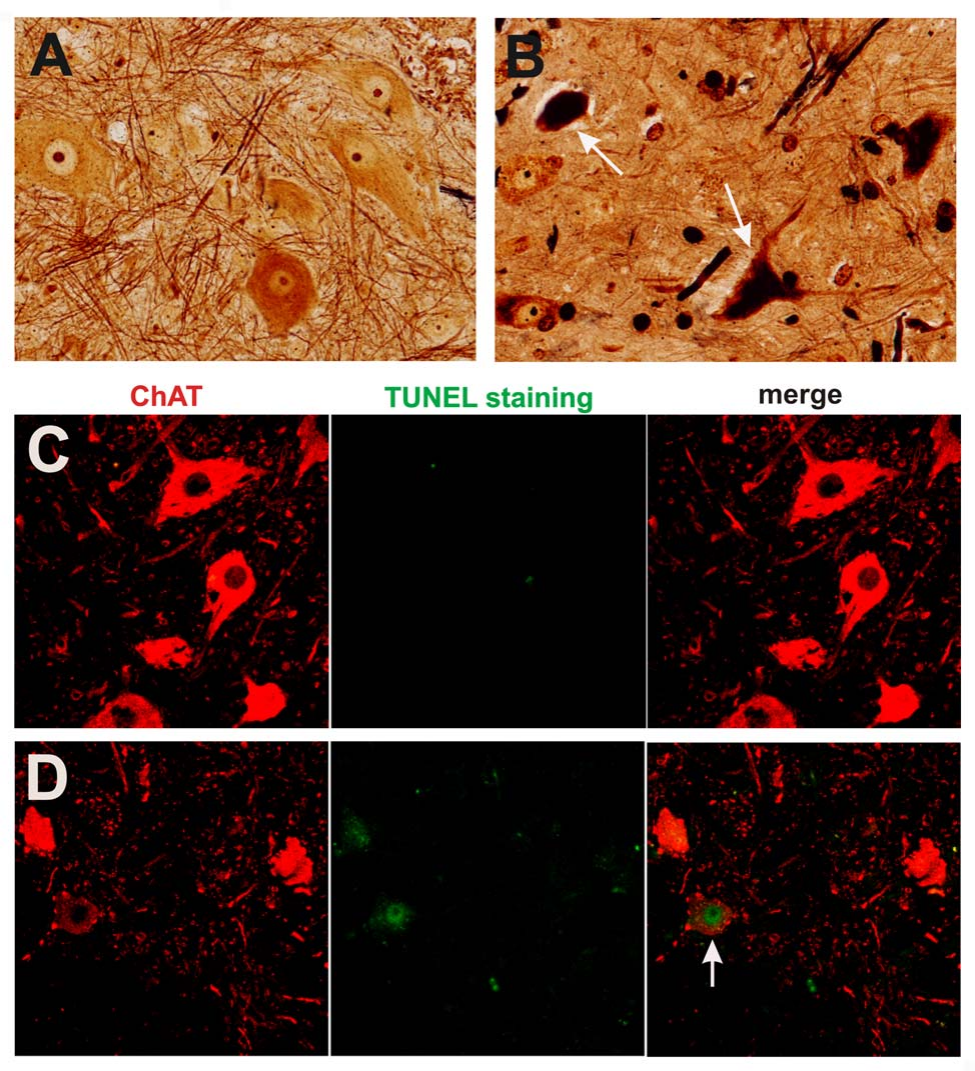
Degeneration of motor neurons in paralyzed mutant *TDP* transgenic rats. (A,B) Bielschowski silver staining revealed degenerating neurons in the lumbar spinal cord of a paralyzed TRE-TDP-43^M337V^ transgenic rat (line 7) (B), but not in the spinal cord of a nontransgenic littermate (A). (C,D) TUNEL staining shows motor neurons in the spinal cord undergoing apoptosis (D: arrow) in a paralyzed TRE-TDP-43^M337V^ transgenic rat ( line 7) (D), but not in its nontransgenic littermate (C). Transverse sections of the L3 spinal segment were stained with ChAT antibody to visualize motor neurons and were labeled using a TUNEL staining kit to visualize apoptosis.

**Figure 6:**
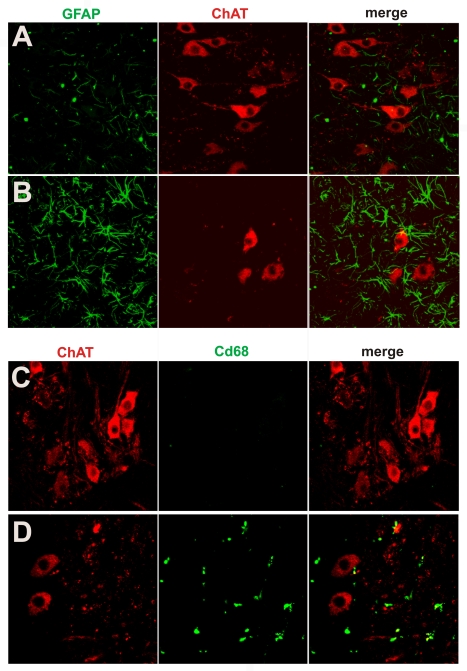
Activation of glial cells in paralyzed mutant *TDP* transgenic rats. (A,B) Double immunofluorescence staining shows an accumulation of astrocytes around motor neurons in a paralyzed TRE-TDP-43^M337V^ transgenic rat (line 7) (B), but not in a nontransgenic littermate (A). (C,D) Double immunofluorescence staining shows an activation of microglial cells in a paralyzed TRE-TDP-43^M337V^ transgenic rat (line 7) (D), but not in a nontransgenic littermate (C). Transverse sections of the L3 spinal cord were immunostained for ChAT (red; motor neuron marker), GFAP (green; astrocyte marker), or cd68 (green; microglia and macrophage marker).

Silver staining revealed that, in mutant *TDP* transgenic rats, spinal motor neurons degenerated during end-stage disease (Figure 5A and 5B). A previous study showed that transient expression of the mutant, but not the normal human *TDP* gene, causes apoptotic death in the spinal cord of chicken embryos [15]. Consistent with the finding from this transient transfection study [15], motor neurons in the spinal cord underwent apoptosis in paralyzed transgenic rats (Figure 5C and 5D). Studies of mutant SOD1 mice suggest that glial cells play an important role in ALS pathogenesis [28], [30]–[33]. Therefore, we examined glial reactions in our paralyzed rats. We found that astrocytes and microglia were increased around the motor neurons in the spinal cord (Figure 6A–6D). The finding suggests that a glial reaction occurs in response to motor neuron degeneration.

### Neurodegeneration is not restricted to motor neurons at end stages of disease

**Figure 7:**
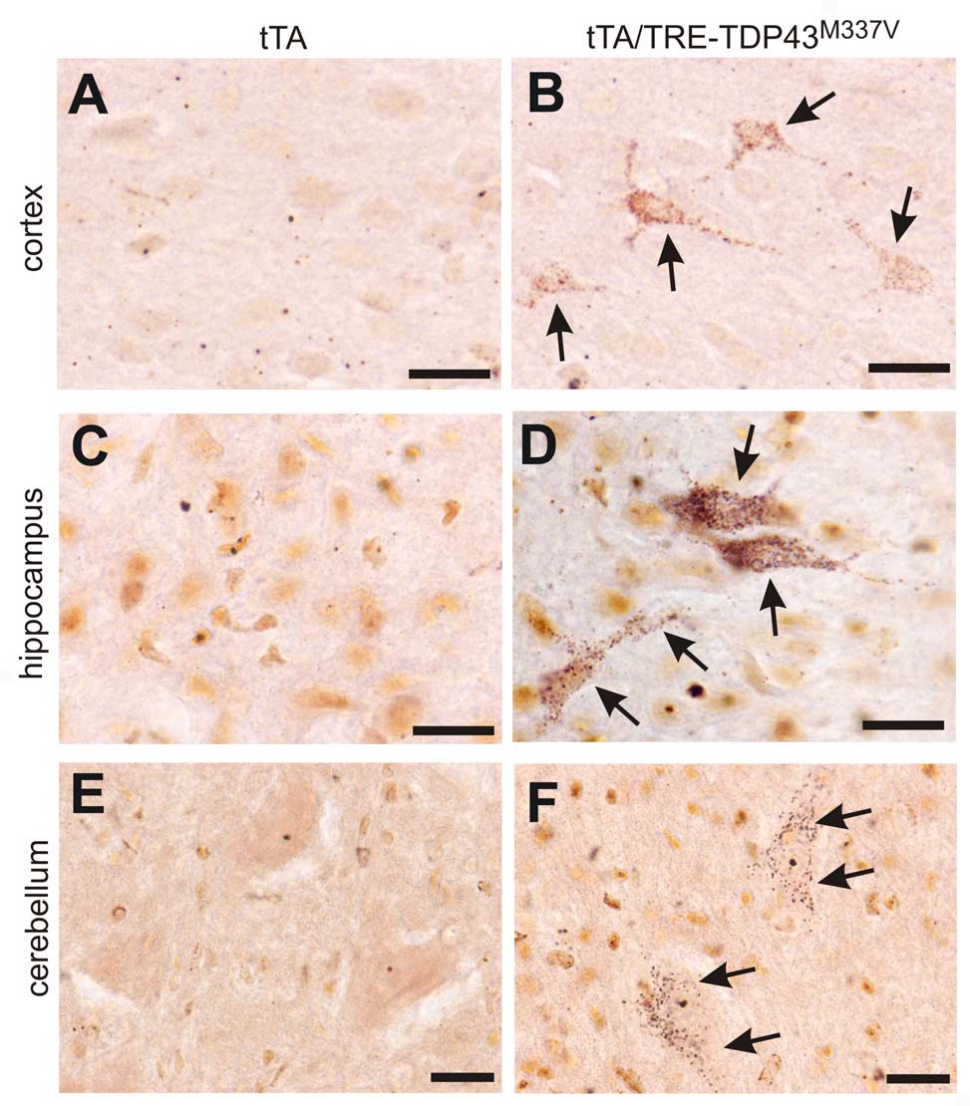
Degeneration of non-motor neurons in paralyzed mutant *TDP* transgenic rats. (A–F) FD silver staining shows degeneration of non-motor neurons in paralyzed tTA/TRE-TDP-43^M337V^ bigenic rats (line 7; age: 45 days) (B,D,F), but not in a tTA transgenic littermate (A,B,C). Degenerating neurons were outlined by deposits of silver particles (arrows). Scale bars: 20 µm.

TDP-43 inclusions are found in the brain and spinal cord of patients with sporadic ALS, FTLD, Alzheimer's disease, or dementia with Lewy bodies [5]–[9], suggesting that TDP-43 proteinopathies are common to neurodegenerative diseases. Pathogenic mutations in the *TDP* gene have been identified not only in ALS, but also in FTLD-MND [14]–[18]. Degeneration associated with mutations in the *TDP* gene may not be restricted to motor neurons. Indeed, silver staining revealed that neurodegeneration occurred in the cortex, hippocampus, and cerebellum of mutant transgenic rats with end stages of disease (Figure 7A–7F) but not in those with earlier stages of disease (Figure 2D and data not shown). Nevertheless, degenerating neurons were not detected in the substantia nigra of paralyzed rats (data not shown), despite the fact that transient overexpression of the normal human *TDP* gene in rats has been shown to induce a loss of dopaminergic neurons in this brain region [34]. Neuropathological findings were correlated with phenotypic expression in mutant *TDP* transgenic rats (Figure 2, Figure 3, Figure 4). Toxicity of the pathogenic *TDP* gene mutation was not restricted to motor neurons, though these neurons were affected by the mutation to a greater degree than all the other neuron types examined.

### Phosphorylated TDP accumulates in affected cells in TDP transgenic rats

**Figure 8:**
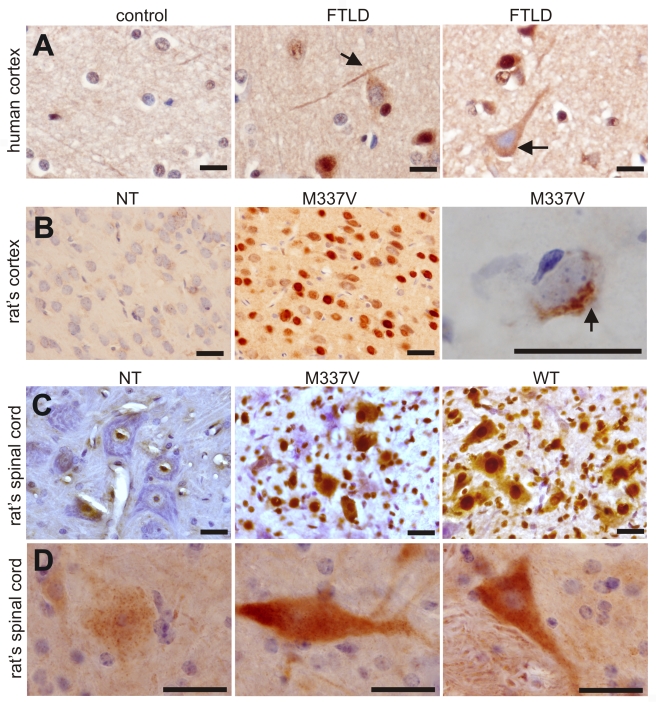
Phosphorylation and cytoplasmic localization of TDP-43 in FTLD patients and in *TDP* transgenic rats. (A) Immunohistochemistry revealed phosphorylation and cytoplasmic localization of TDP-43 (arrows) in the brains of FTLD patients, but not in control subjects. Paraffin-embedded sections were stained with a polyclonal antibody against TDP-43 phosphorylated at aa409/410. (B,C) Immunostaining revealed that human TDP-43 was present in both the nucleus and cytoplasm of cells from the mutant (M337V: line 7) and normal (WT) *TDP* transgenic rats, but not in the cells of a nontransgenic rat (NT). TDP-43 inclusions (arrow) were rarely detected, being present in only the cortex of paralyzed M337V transgenic rats. Cryopreserved sections of the rat brain and spinal cord were stained with a polyclonal antibody specific to human TDP-43. (D) Immunostaining shows a cytoplasmic accumulation of phosphorylated TDP-43 in mutant (M337V) and normal (WT) *TDP* transgenic rats. A weak signal for phosphorylated TDP-43 was also detected in the nuclei of cells from a nontransgenic rat (NT). Cryopreserved sections of the spinal cord were stained with a polyclonal antibody against TDP-43 phosphorylated at aa409/410. All scale bars: 20 µm.

Phosphorylated TDP-43 inclusions are a signature pathological feature of sporadic ALS and FTLD [5], [6], [35]–[37]. To detect phosphorylated TDP-43 inclusions in our transgenic rats, we tested a polyclonal antibody specific to phosphorylated TDP-43 on brain sections of FTLD patients and *TDP* transgenic rats. This phospho-TDP-43 antibody detected cytoplasmic accumulation of phosphorylated TDP-43 in FTLD patients, but not in control subjects (Figure 8A). Similarly, phosphorylated TDP-43 was diffusely distributed in affected neurons in transgenic rats expressing the mutant or normal human *TDP* transgene (Figure 8D). We generated a polyclonal antibody recognizing both phosphorylated and non-phosphorylated human TDP-43 (Figure 1C–1F) and detected a robust expression of the human *TDP* transgene in transgenic rats (Figure 8B and 8C). TDP-43 was diffusely distributed in the nucleus and cytoplasm of cells within transgenic rats (Figure 8). However, TDP-43 inclusions were detected rarely, being present only in the cortex (Figure 8B) and not in the spinal cord (Figure 8C) of transgenic animals.

Immunohistochemistry revealed that typical ubiquitin-positive inclusions were not present in the spinal cords of normal or mutant *TDP* transgenic rats, though the intensity of ubiquitin immunostaining was greater in these animals than in nontransgenic rats (Figure S4). Since TDP-43 inclusions were rare in transgenic rats, even at end-stage disease, we further examined TDP-43 ubiquitination using immunoprecipitation combined with immunoblotting analysis. Ubiquitinated TDP-43 was detected in the mutant *TDP* transgenic rats (Figure S4). Immunoblotting revealed that a small amount of TDP-43 fragments (less than 43 kDa) was present in *TDP* transgenic rats (Figure 2B and Figure S5). TDP-43 fragments were detected in urea tissue extracts from rats at the paralysis stage, but not in extracts from those at disease onset (Figure S5). The finding suggests that the solubility of the small TDP-43 fragment is reduced as the disease progresses.

## Discussion

Expression of the human *TDP* gene containing a M337V substitution reproduced the phenotypes of ALS in rats. That is, these animals exhibited progressive degeneration of motor neurons and denervation atrophy of skeletal muscles. In this transgenic rat model, neurodegeneration was not restricted to motor neurons and could be seen in other types of neurons including cortical neurons, hippocampal neurons, and cerebellar neurons. However, *TDP* mutation affected motor neurons earlier and more severely than other neurons in the central nervous system (CNS). This robust rat model also recapitulated features of TDP-43 proteinopathies, including the formation of TDP-43 inclusions, cytoplasmic localization of phosphorylated TDP-43, and fragmentation of TDP-43.

While our transgenic rat model developed the phenotypes of ALS, it displayed degeneration of CNS neurons other than motor neurons at the end stages of the disease. Our findings in mutant *TDP* transgenic rats do not necessarily contradict observations in ALS patients. ALS is traditionally thought to affect only motor neurons, but recent studies showed that neurons other than motor neurons also degenerate in ALS [38]. This point is strikingly illustrated by the observation in some ALS patients who live with the disease much longer than the average disease duration [38]–[40]. Moreover, some ALS and FTLD cases share symptoms and pathological characteristics [41]. Although mutations of the *TDP* gene are primarily associated with ALS [14]–[17], a recent study found that a novel mutation in the *TDP* gene is associated with FTLD-MND [18], suggesting that the toxicity (if any) of the *TDP* gene mutation is not restricted to motor neurons [18]. Further studies are warranted to ascertain whether a correlation exists between the pathological changes induced by *TDP* mutation and TDP-43 proteinopathies observed in sporadic ALS and FTLD. The fact that TDP-43 proteinopathy is observed in a wide range of neurodegenerative diseases suggests that mutations in the *TDP* gene are generally neurotoxic [5], [6], [9], [42]–[45]. Neurodegenerative diseases may share common pathological mechanisms, with a certain subgroup of neurons being predominantly affected under each disease condition. Our mutant *TDP* transgenic rat is a robust model of neurodegeneration caused by mutation of the *TDP* gene.

Many features of TDP-43 proteinopathies were reproduced in our *TDP* transgenic rats. Redistribution, phosphorylation, and aggregation of TDP-43 are all hallmarks of sporadic FTLD and ALS [5],[44],[45]. A recent clinical study showed that TDP-43 redistribution appears to be an early event in TDP-43 proteinopathy [7], suggesting that TDP-43 redistribution underlies the pathogenesis of neurodegeneration. Our results showed that phosphorylated TDP-43 was diffusely distributed in the cytoplasm and nucleus of affected cells in paralyzed mutant *TDP* transgenic rats as well as in non-paralyzed, normal *TDP* transgenic rats. The presence of phosphorylated TDP-43 in normal *TDP* transgenic rats does not exclude the possibility that TDP-43 phosphorylation contributes to pathogenesis induced by *TDP* mutation. Specifically, *TDP* mutation may impart toxicity by enhancing the normal functions of the gene. For example, mutation of the *LRRK2* gene causes Parkinson's disease by enhancing (at least partially) the kinase activity of LRRK2 [46],[47]. Gene mutations can be classified into three types based on their effect on protein function: gain of function, loss of function, and dominant negative effect. Pathogenic mutation of the *TDP* gene may cause disease through any one of these three effects on protein function. Resolving the nature of the *TDP* gene mutation will require a more sophisticated model such as a knockin mouse. TDP-43 inclusions and fragmentation were rarely observed and were present only at end-stage disease, suggesting that these pathologies may be consequence of, rather than a cause of, neurodegeneration in *TDP* transgenic rats. C-terminal truncated products of TDP-43 are thought to result from caspase cleavage of full-length TDP-43 [48]. Accordingly, C-terminal fragmentation of TDP-43 is likely a consequence, instead of a cause, of neurodegeneration because caspase activation is a terminal feature of cell death. In addition, we cannot exclude the possibility that overexpression of the *TDP* transgene interferes with rat development, since the mutant *TDP* transgenic rats died at postnatal ages.

Typical ALS has a late onset and rapidly progresses [12], [13], [17], [29], [49]–[51]. In contrast, mutant *TDP* transgenic rats developed paralysis at early ages, with the paralysis being similar to that seen in ALS. Early onset of disease phenotypes in our rat model likely results from toxicity of the *TDP* gene mutation, as evidenced by the following three findings. First, paralysis and lethality were observed in the mutant miniTDP43^M337V^ transgenic founders, but not in the normal miniTDP43^WT^ transgenic founders. Second, paralysis and neurodegeneration were observed in the inducible mutant *TDP* transgenic rats, but not in offspring of the constitutive normal *TDP* transgenic rats, despite the fact that both lines exhibited comparable expression of the human *TDP* transgenes. Third, similar phenotypes were observed in the constitutive mutant *TDP* transgenic founders and in the inducible mutant *TDP* transgenic offspring. One transgenic founder rat carried only six copies of the mini mutant *TDP* transgene and developed paralysis in postnatal age. The copy number of the mutant *TDP* transgene that is required for phenotypic expression in transgenic rats is much lower than the copy threshold of mutant *SOD1* transgenes [52],[53]. To activate the inducible mutant *TDP* transgene, we used a low-copy tTA transgenic line that carries only two copies of the tTA transgene [24]. Therefore, expression levels of the *TDP* transgene in the inducible transgenic rats were comparable to those in the constitutive normal TDP transgenic rats. Transgenic rats expressing the mutant *TDP* gene displayed a wider range of neurodegeneration than transgenic rodents expressing mutant *SOD1* genes [29], [52]–[54], with neurodegeneration predominantly affecting the motor system. Such unrestricted toxicity of the *TDP* gene mutation may lead to an early onset of the disease. In some aspects, phenotypes observed in our transgenic rats are similar to those detected in transgenic mice that express the human *TDP* gene with a A315T substitution [55]. In these rodent models, both upper and lower motor neurons are affected and TDP-43 inclusions are rare. However, our rat model developed paralysis at postnatal ages and experienced a rapid disease progression, while the mutant *TDP* transgenic mice develop disease phenotypes during middle age and have varying disease durations [55]. Different mutations in the *TDP* gene and different animal species may contribute to phenotypic variation between the rat and mouse models.

Our findings in *TDP* transgenic rats indicate that mutation of the *TDP* gene is highly toxic in rodents, though the nature of the pathogenic mutation in the *TDP* gene remains to be resolved. Since deletion of the *TDP* gene in *Drosophila* causes defects at NMJs [19], the possibility that the *TDP* gene mutation produces a dominant-negative effect cannot be excluded. Although the nature of *TDP* gene mutation will need to be determined using a more sophisticated model, our *TDP* transgenic rats will be useful for mechanistic study of TDP-43-related neurodegenerative diseases.

## Materials and Methods

### Ethics statement

Animal use followed NIH guidelines. The animal use protocol was approved by the Institutional Animal Care and Use Committees (IACUC) at Thomas Jefferson University. The Committee for Oversight of Research Involving the Dead at the University of Pittsburgh School of Medicine approved the use of human tissue from the University of Pittsburgh ALS Tissue Bank. Age-matched tissue sections from two FTLD and two non-neurological disease controls were used for the study.

### Transgene constructs

The 22-kb mini human *TDP* gene was extracted from a BAC clone (RP11-829B14), and a M337V substitution was introduced into the mini *TDP* gene by homologous recombination in *Escherichia coli* [22]. The normal and mutant mini TDP transgenes were linearized by restriction digestion, purified from agarose gels, and then used to produce transgenic rats through microinjection. To generate Tet-regulatable *TDP* transgenic rats, we PCR-amplified the human TDP-43 ORF from a human brain cDNA pool (Invitrogen) and generated a mutant carrying the M337V substitution using site-directed mutagenesis (Stratagene). The mutated human TDP-43 cDNA gene was inserted downstream of a tTA-dependent promoter that was constructed by fusing seven tetracycline-responsive elements (TRE) with a minimal cytomegalovirus promoter (TRE-miniCMV). To enhance gene splicing and expression, we inserted the first intron of the human ubiquitin C gene between the TRE-miniCMV promoter and the TDP-43 ORF [24].

### Transgenic animal production

Linearized miniTDP43 and TRE-TDP43 transgenes were injected into the pronuclei of fertilized eggs of Sprague-Dawley rats. The injected eggs were then transplanted into pseudopregnant females for embryonic development [56]. Transgenic founders carrying miniTDP-43 transgenes were analyzed for disease phenotypes. Transgenic founders carrying TRE-TDP-43 transgenes were crossed with CAG-tTA transgenic rats to produce double transgenic offspring, which were analyzed for transgene expression and disease phenotypes. The *TDP* transgenic rats were identified by PCR amplification of the human *TDP* gene using the following primer pair: 5′-TGCGGGAGTTCTTCTCTCAG (forward) and 5′-AGCCACCTGGATTACCACCA (reverse). The copy number of the transgene was determined by quantitative PCR using two primer pairs. The first primer pair was designed to amplify a DNA fragment of the same composition from both the human and the rat *TDP* gene: 5′-TGAGCCCATTGAAATACCATC-3′ and 5′-TACACTGAGACACTGGATTC. The second primer pair was designed to amplify the rat prolactin gene as an internal control: 5′-CCTCTATGAACGAAACCCAC-3′ and 5′-CTTCCGGCTAATCCA CAATG-3′.

### Antibody production

A rabbit polyclonal antibody was produced by Genemed Company. Rabbits were immunized with the synthetic peptide, (N-terminal)-EDELREFFSQYGDVM. Antiserum was then affinity-purified using a peptide-conjugated column (Pierce).

### Immunohistochemistry

Under deep anesthesia, animals were transcardially perfused with 1X PBS (pH 7.4) and then with 4% paraformaldehyde (PFA) dissolved in 1X PBS buffer. The brain, spinal cord, and gastrocnemius muscle of perfused animals were collected and further fixed in the same fixative overnight. Some tissue blocks were embedded in paraffin and sectioned into 10 µm-thick slices. Paraffin-embedded sections were treated with 10 mM sodium citrate buffer (pH 6.0) to retrieve antigens for immunostaining. Paraffin-embedded coronal sections of the brain and transverse sections of the spinal cord were deparaffinized and immunostained with human TDP-43-specific antibody (1∶1,000; made in house) or a phospho-TDP-43-specific antibody (1∶1,000; COSMO Bio Co., TIP-PTD-P02). Immunostaining was visualized using an ABC kit in combination with diaminobenzidine (Vector). The immunostained sections were lightly counterstained with hematoxylin to display nuclei. After antigen retrieval, paraffin-embedded sections of the lumbar spinal cord were immunostained for human TDP-43 (1∶300) and ChAT (goat antiserum; Millipore). Immunofluorescence staining for human TDP-43 (red) and ChAT (green) was visualized using a Nikon fluorescence microscope, and images were acquired using a Nikon digital camera. Paraffin-embedded sections of the gastrocnemius muscle were stained with hematoxylin and eosin (H&E) to visualize tissue structures.

For NMJ detection, gastrocnemius muscles were fixed in 4% PFA for 2 h and sectioned on a cryostat into 100 µm-thick sections. Serial sections of the muscles were incubated with α-bungarotoxin (Invitrogen) for 30 min, washed in PBS three times, incubated overnight with mouse monoclonal antibodies to neurofilament (Sigma) and synaptophysin (Millipore), and then incubated for 1 h with a secondary antibody (FITC goat anti-mouse IgG1; Jackson Immunology).

For detection of apoptotic cells and glial cells, 4% PFA-fixed lumbar spinal cords were cut into three sets of 10 µm-thick serial sections on a cryostat. Every first section was incubated with TUNEL staining reagent (Millipore) and goat anti-ChAT antibody. Every second section was incubated with the ChAT antibody and mouse anti-GFAP. Every third section was incubated with the ChAT antibody and mouse anti-CD68 antibody. Sections were then incubated with appropriately labeled secondary antibodies. The antibodies were purchased from Millipore. Images were captured using a Zeiss LSM510 META confocal system. The NMJ was reconstructed using *z*-stack projections produced from serial scanning every 1 µm.

### Esterase histochemistry

Fresh gastrocnemius muscle was snap-frozen in liquid nitrogen and cut into 12 µm-thick sections on a cryostat. Nonspecific esterase activity was detected using the α-napthyl acetate protocol. Denervated muscle fibers were stained a red-brown color, with normal fibers displaying a yellow-to-brown color.

### Silver staining

Degenerating neurons were visualized using the Bielschowski silver-staining method as well as the FD NeuroSilver kit (FD Neurotechnologies, Baltimore, MD). For the Bielschowski silver method, paraffin-embedded spinal cord was transversely cut into 10 µm-thick sections. For staining using the FD NeuroSilver kit, 40 µm-thick coronal sections were obtained by slicing through the forebrain and cerebellum using a cryostat and then stained per the manufacturer's instructions.

### Toluidine blue staining and electron microscopy

Rats were anesthetized and perfused with a mixture of 4% PFA and 2% glutaraldehyde in 0.1 M phosphate buffer (pH 7.4). The L3 and L4 ventral and dorsal roots were removed and post-fixed in the same fixative at 4°C overnight. The roots were then further fixed in 1% osmium tetroxide in 0.1 M phosphate buffer (pH 7.4) for 1 h. The well-fixed tissues were dehydrated in graded ethanol and embedded in Epon 812 (Electron Microscopic Sciences, Fort Washington, PA). Thin sections (80 nm) were then stained with uranyl acetate and lead citrate for observation under a transmission electron microscope (Hitachi H7500-I). For toluidine staining, roots were transversely cut into 1 µm-thick sections. Axons in the nerve roots were examined in the semi-thin sections under a light microscope (Olympus AX70).

### Cresyl violet staining and stereological cell counting

A 1-mm central segment of the L3 spinal cord was cut into 30-µm thick sections using a cryostat. Every third section was stained with cresyl violet and mounted in sequential order (rostral-caudal). Neurons with a diameter larger than 25 µm were counted in the ventral horns on both sides. The number of targeted neurons was estimated using a fractionator-based unbiased stereology software program (Stereologer), which was run on a PC computer that was attached to a Nikon 80i microscope with a motorized XYZ stage (Prior). At low magnification (40x), the targeting area was outlined, and a random sampling grid was created. At 1000× magnification, an optical dissector probe was randomly generated by the program in the designated area. The presence of clearly definable neurons was noted according to defined inclusion and exclusion limits of the dissector. This process was repeated on all selected sections. The total number of defined neurons was calculated by the software based on values obtained from random counts.

### Electromyography

Animals were anesthetized during electromyography (EMG) examination. The fibrillation potential of the gastrocnemius muscle was recorded with an EMG instrument (CMS6600; COTEC Inc.) using a 27-gauge monopolar needle electrode and a 29-gauge reference needle electrode. During recording, a sub-dermal ground electrode was placed in the forelimb. Spontaneous electrical activity of selected skeletal muscle was recorded for 2 min.

### Statistical analysis

The number of defined neurons in the ventral horn was compared between groups of transgenic rats. The difference in the number of neurons was analyzed using an unpaired t test. The null hypothesis was rejected at the 0.05 level.

## Supporting Information

Figure S1 Recovery of *TDP* transgene expression after Dox withdrawal. Breeding rats of TRE-TDP-43^M337V^ transgenic line 16 were constantly given Dox in drinking water (50 ug/ml) and the pregnant female rats were deprived of Dox four days before delivery. Forebrain of the offspring doubly transgenic for the tTA and the TRE-TDP-43^M337V^ was dissected at varying ages. Western blotting detected a robust expression of the *TDP* transgene in the offspring by age of 5 days when a human TDP-43-specific antibody was used to detect TDP-43 immunoreactivity. Each lane was loaded with 20 µg of total protein in brain lysate. Immunoreactivity of GAPDH was detected as an internal control for equal loading.
(0.59 MB TIF)

Figure S2 Axons of dorsal root affected in the TRE-TDP-43^M337V^ transgenic rats at paralysis stage. (A,B) Toluidine blue staining shows axons of the L3 dorsal roots taken from the tTA/TRE-TDP-43^M337V^ double (B) or a age-matched tTA single (A) transgenic rat. (C,D) EM shows axons in the dorsal root of the tTA/TRE-TDP-43^M337V^ double (D) or a age-matched tTA single (C) transgenic rat. The mutant rat was terminated when its two legs paralyzed at the age of 45 days. L3 nerve root was dissected for histology. Affected axon was shrunk with collapsed myelin (arrow). Scale bars: 2 µm.
(9.52 MB TIF)

Figure S3 Degeneration of dorsal corticospinal tract in the TRE-TDP-43^M337V^ transgenic rats at paralysis stage. (A,B) Toluidine blue staining revealed degeneration of motor axons in the dorsal corticospinal track of the tTA/TRE-TDP-43^M337V^ double transgenic rat (B), but not in its tTA single transgenic littermate (A). Low cervical spinal cord was dissected from a paralyzed TRE-TDP-43^M337V^ transgenic rat or its tTA transgenic littermate. Arrows point to some dilated axons in the dorsal corticospinal track of spinal cord.
(8.97 MB TIF)

Figure S4 Ubiquitination of TDP-43 in *TDP* transgenic rats. (A–C) Micrographs of spinal ventral horns show ubiquitin Immunohistochemistry on transverse sections of L3 spinal cords taken from an age-matched nontransgenic rat (A), a miniTDP-43^WT^ transgenic rat of line 4 (B), or a paralyzed TRE-TDP-43^M337V^ transgenic rat of line 7 (C). Tissue sections were lightly counterstained with haematoxylin to show cell nuclei. Note no typical ubiquitin-positive inclusion in the wildtype and mutant *TDP* transgenic rats though the intensity of ubiquitin immunostaining was relatively enhanced in *TDP* transgenic rats (B,C) compared to nontransgenic control (A). (D,E) Immunoprecipitation in combination with immunoblotting revealed ubiquitination of TDP-43 (D), but not FUS (E), in the mutant *TDP* transgenic rats with paralysis. Urea extracts of rat's brain were immunoprecipitated with antibodies to TDP-43 or FUS and the precipitants were further analyzed by immunoblotting for ubiquitin (MAB1510: Millipore) and TDP-43 or FUS immunoreactivity.
(8.94 MB TIF)

Figure S5 Fragmentation of TDP-43 in *TDP* transgenic rats with end-stage disease. Immunoblotting with an antibody to the C-terminal of TDP-43 detected fragments of TDP-43 in the mutant transgenic rats at paralysis stage (P), but not at disease onset (O). SC: spinal cord. Urea extracts of rat's tissues were resolved on 12% SDS-PAGE and transferred onto membrane. The membrane was first probed with the TDP-43 antibody and subsequently probed with a GAPDH antibody.
(0.62 MB TIF)
