# Supplementary figures and images for: Transgenic Rat Model of Neurodegeneration Caused by Mutation in the TDP Gene
Source: PLoS Genet. 2010 Mar 26;6(3):e1000887. doi: 10.1371/journal.pgen.1000887 (PMC2845661; doi:10.1371/journal.pgen.1000887)

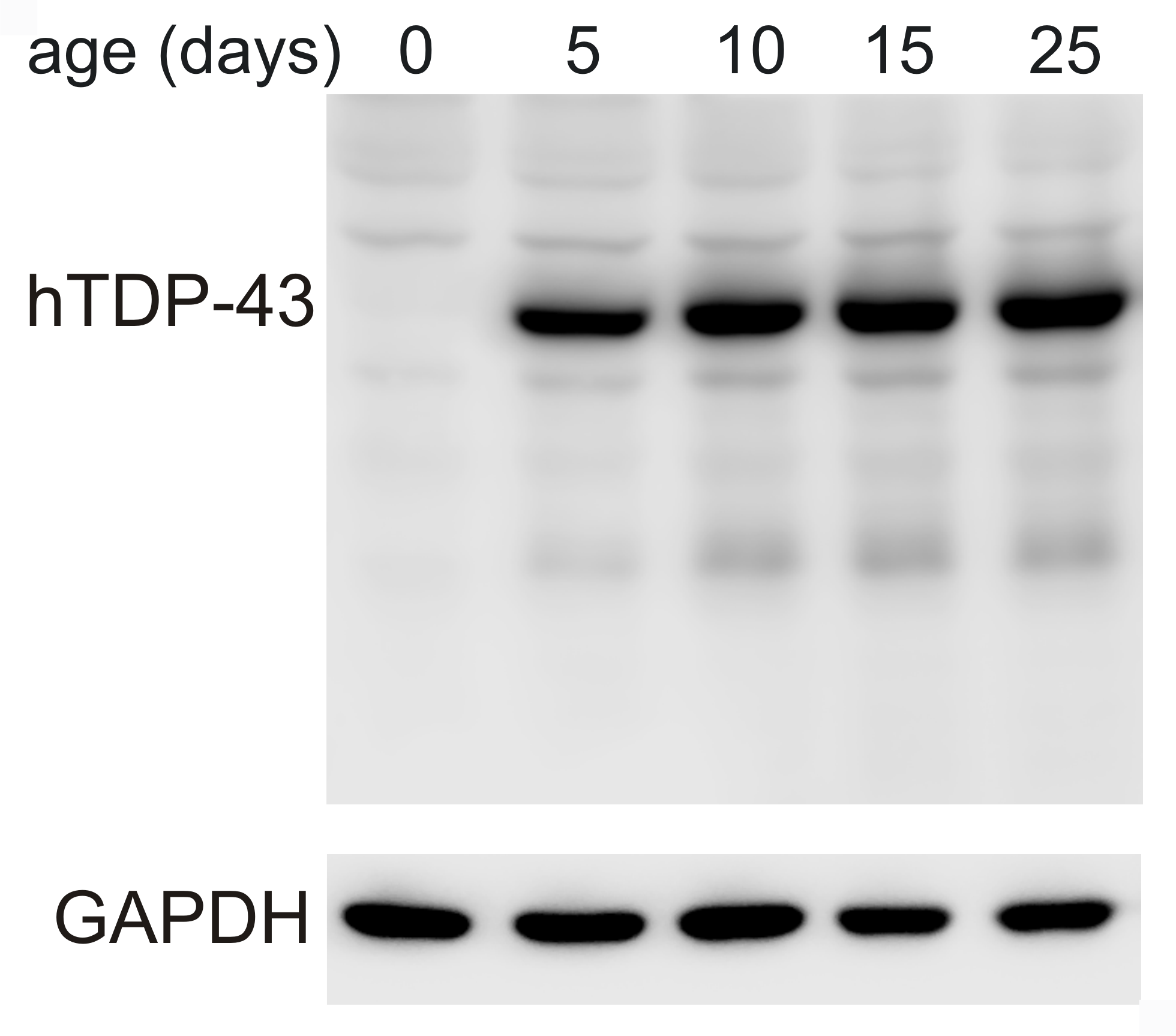

Supplement: Figure S1 — Recovery of TDP transgene expression after Dox withdrawal. Breeding rats of TRE-TDP-43M337V transgenic line 16 were constantly given Dox in drinking water (50 ug/ml) and the pregnant female rats were deprived of Dox four days before delivery. Forebrain of the offspring doubly transgenic for the tTA and the TRE-TDP-43M337V was dissected at varying ages. Western blotting detected a robust expression of the TDP transgene in the offspring by age of 5 days when a human TDP-43-specific antibody was used to detect TDP-43 immunoreactivity. Each lane was loaded with 20 µg of total protein in brain lysate. Immunoreactivity of GAPDH was detected as an internal control for equal loading. (0.59 MB TIF) [file pgen.1000887.s001.tif]

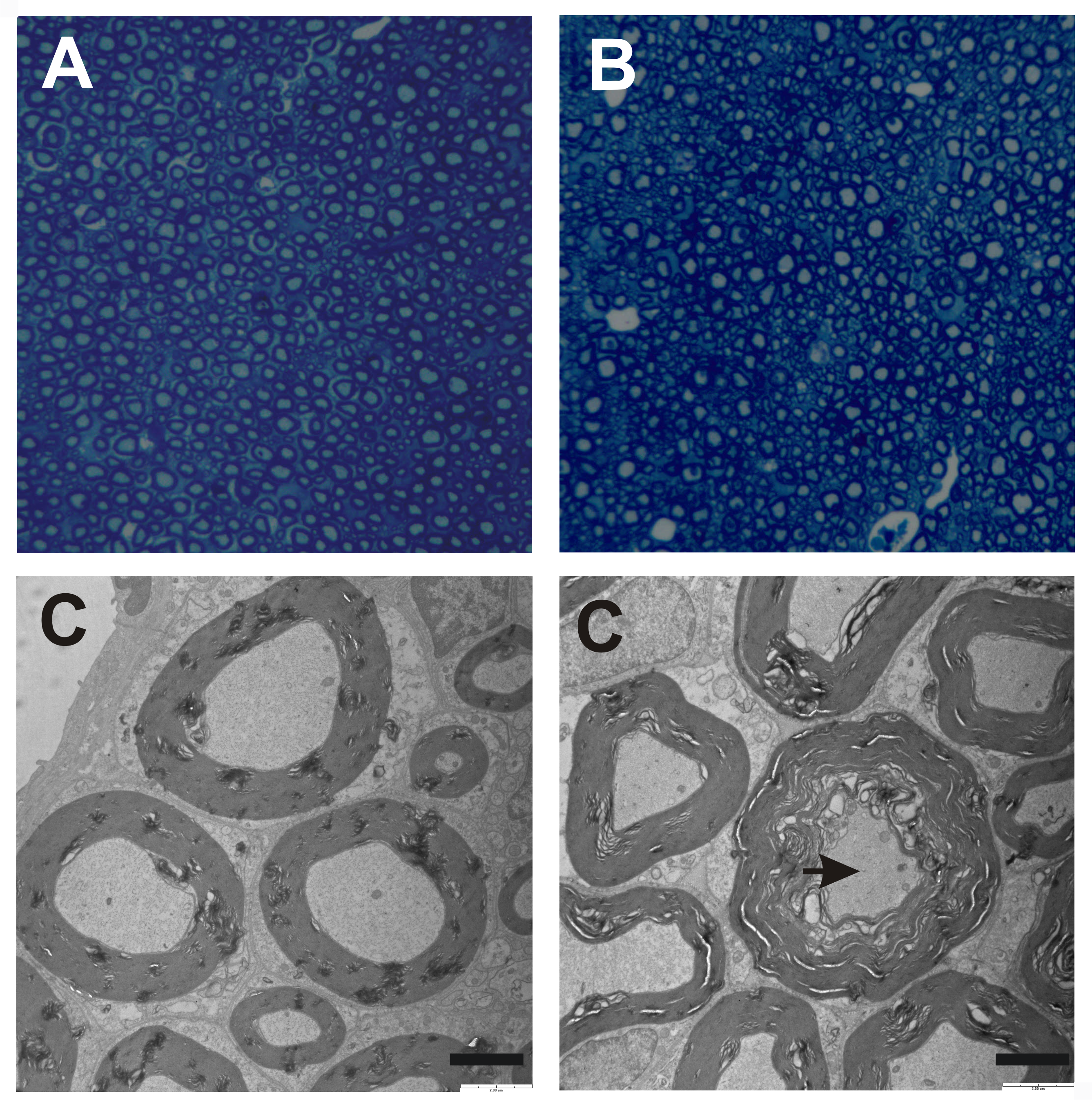

Supplement: Figure S2 — Axons of dorsal root affected in the TRE-TDP-43M337V transgenic rats at paralysis stage. (A,B) Toluidine blue staining shows axons of the L3 dorsal roots taken from the tTA/TRE-TDP-43M337V double (B) or a age-matched tTA single (A) transgenic rat. (C,D) EM shows axons in the dorsal root of the tTA/TRE-TDP-43M337V double (D) or a age-matched tTA single (C) transgenic rat. The mutant rat was terminated when its two legs paralyzed at the age of 45 days. L3 nerve root was dissected for histology. Affected axon was shrunk with collapsed myelin (arrow). Scale bars: 2 µm. (9.52 MB TIF) [file pgen.1000887.s002.tif]

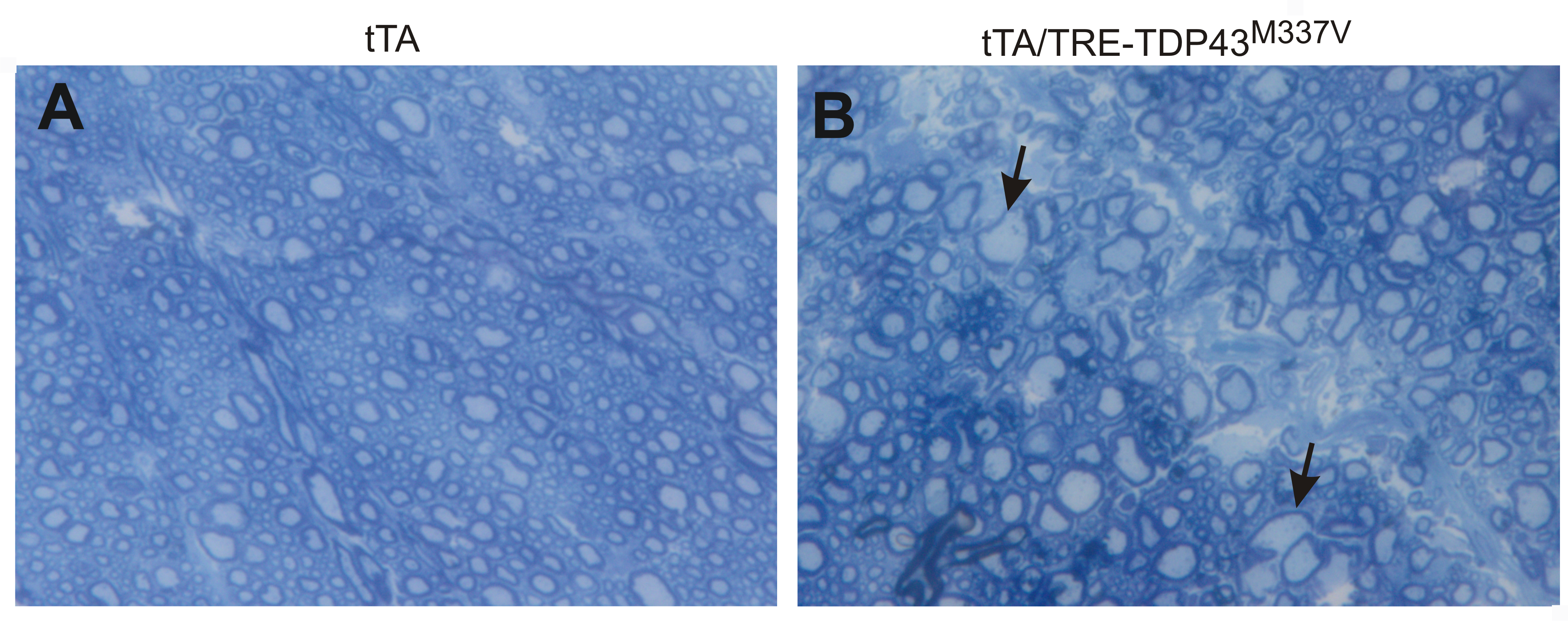

Supplement: Figure S3 — Degeneration of dorsal corticospinal tract in the TRE-TDP-43M337V transgenic rats at paralysis stage. (A,B) Toluidine blue staining revealed degeneration of motor axons in the dorsal corticospinal track of the tTA/TRE-TDP-43M337V double transgenic rat (B), but not in its tTA single transgenic littermate (A). Low cervical spinal cord was dissected from a paralyzed TRE-TDP-43M337V transgenic rat or its tTA transgenic littermate. Arrows point to some dilated axons in the dorsal corticospinal track of spinal cord. (8.97 MB TIF) [file pgen.1000887.s003.tif]

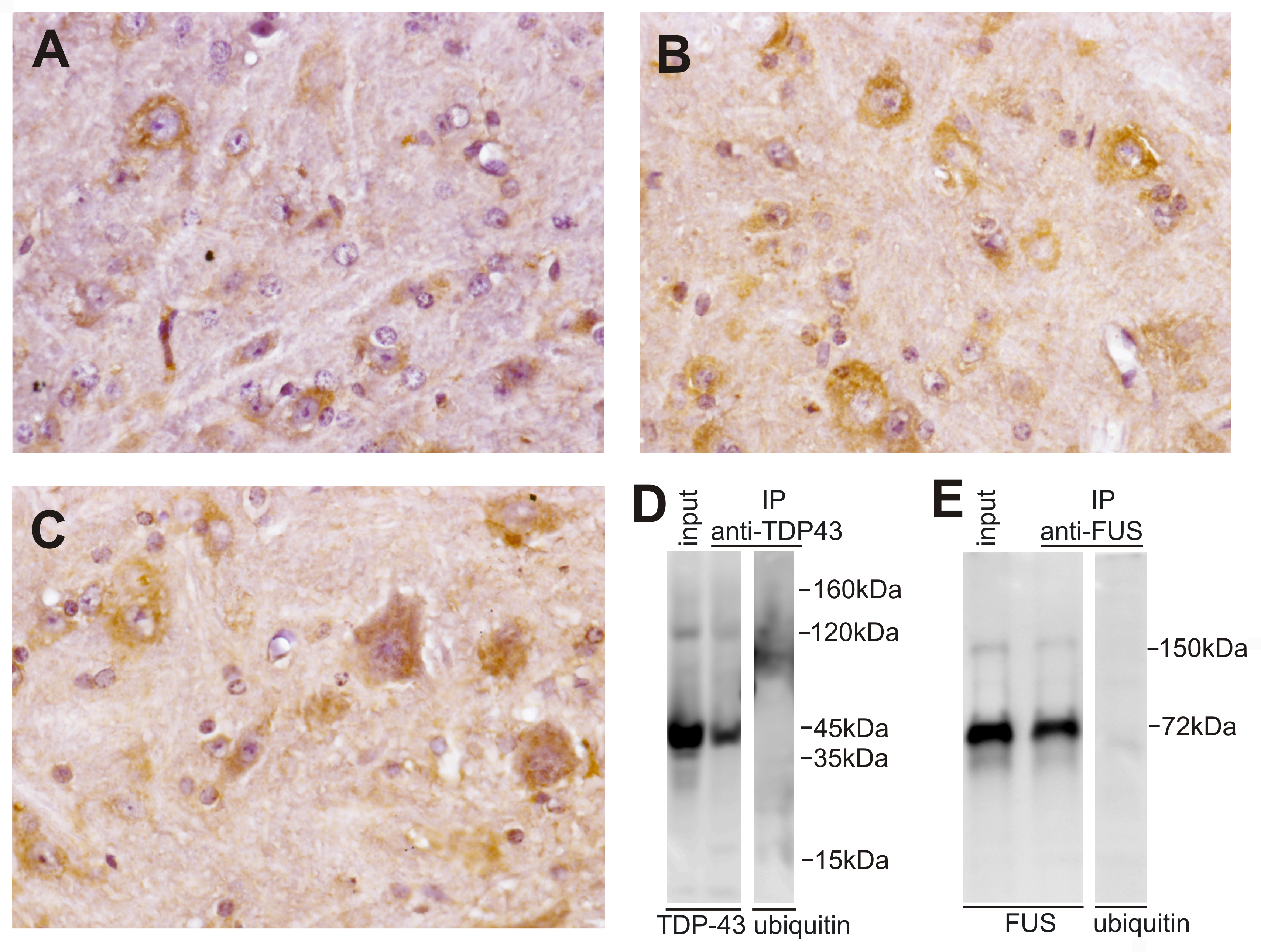

Supplement: Figure S4 — Ubiquitination of TDP-43 in TDP transgenic rats. (A–C) Micrographs of spinal ventral horns show ubiquitin Immunohistochemistry on transverse sections of L3 spinal cords taken from an age-matched nontransgenic rat (A), a miniTDP-43WT transgenic rat of line 4 (B), or a paralyzed TRE-TDP-43M337V transgenic rat of line 7 (C). Tissue sections were lightly counterstained with haematoxylin to show cell nuclei. Note no typical ubiquitin-positive inclusion in the wildtype and mutant TDP transgenic rats though the intensity of ubiquitin immunostaining was relatively enhanced in TDP transgenic rats (B,C) compared to nontransgenic control (A). (D,E) Immunoprecipitation in combination with immunoblotting revealed ubiquitination of TDP-43 (D), but not FUS (E), in the mutant TDP transgenic rats with paralysis. Urea extracts of rat's brain were immunoprecipitated with antibodies to TDP-43 or FUS and the precipitants were further analyzed by immunoblotting for ubiquitin (MAB1510: Millipore) and TDP-43 or FUS immunoreactivity. (8.94 MB TIF) [file pgen.1000887.s004.tif]

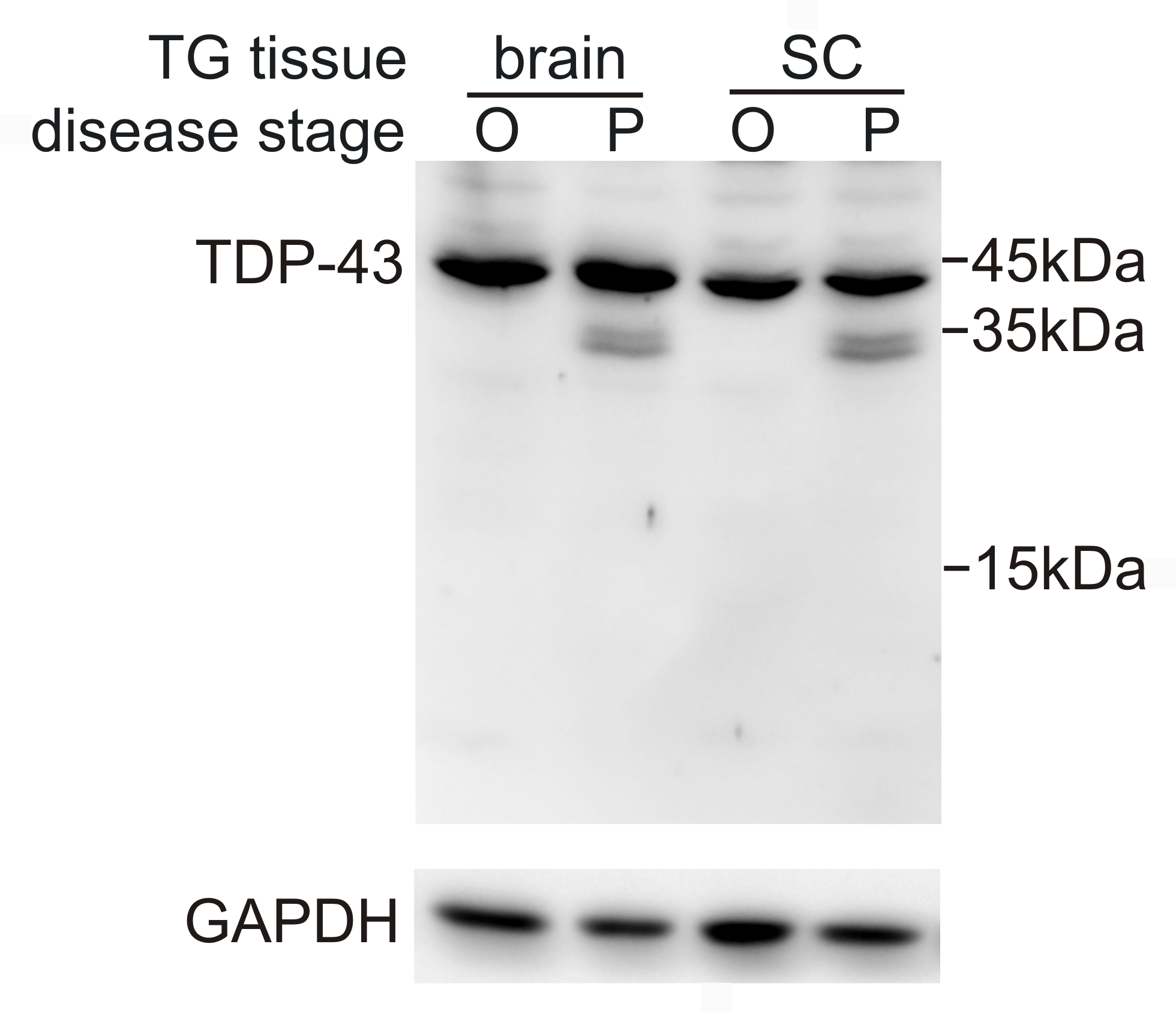

Supplement: Figure S5 — Fragmentation of TDP-43 in TDP transgenic rats with end-stage disease. Immunoblotting with an antibody to the C-terminal of TDP-43 detected fragments of TDP-43 in the mutant transgenic rats at paralysis stage (P), but not at disease onset (O). SC: spinal cord. Urea extracts of rat's tissues were resolved on 12% SDS-PAGE and transferred onto membrane. The membrane was first probed with the TDP-43 antibody and subsequently probed with a GAPDH antibody. (0.62 MB TIF) [file pgen.1000887.s005.tif]
